# Alcohol

**Published:** 1888-05

**Authors:** 


### ALCOHOL.

We extract the following from a recent article by T. B. Wakeman, Esq., published in the *Bureau Record.* It gives some points of interest to the imbibers of alcoholic poison.

It is now known that what we call fermentation is caused by the sudden increase, by millions on millions, of a little animal cell, or microbe, only visible under the microscope, and which used to be called the yeast plant, but which is now dignified by the scientific name of *Torvula Cerevisiae*, which is Latin for the strings or *twists* that appear in cereal ferments, that is, those made from corn or grain, the gift of the old goddess *Ceres.* This little fellow is one of the most wonderful, and, if rightly used, one of the most useful inhabitants of our globe. He is not a plant, as was at first believed, for he feeds only on a vegetable substance, viz : grape or fruit sugar, called in chemistry *glucose*, and he, in so doing, gives off carbonic acid gas, and an *excrement*, which is a substance wholly devitalised by the living process of the little animal, much as ashes result from the burning of coal in a stove. These breathings and excretions only result from animal life, and in every way this microbe assimilates his sugar-food, propagates, breathes, excretes and lives like an animal; and when dead and cremated, smells just like burning animal tissue. Now this *excretion* of this fruit-sugar-eating animal is *alcohol.* All of the alcohol in the world comes from this animal in just this way, and in none other. It is always the same substance, and has always the same properties—just as salt is always salt.

These little microbes, or yeast-animals, are in the air and pretty much everywhere. They dry up and seem to be dead, and float about, but as soon as grape or fruit sugar (glucose) is exposed, they are there, and the rate of their increase in it is marvelous. He is the father of our bread as well as of our wine. We know him best as the active agent in the yeast cake, whence comes the useful fermentation which lightens our bread previous to baking. A penny yeast cake, dry as a chip, contains at least, 7,000,000 of these animals. Put in a warm dough, and in an hour he will count over 140,000,000! and this increase and his minute carbonic acid gas-breaths will have made the dough *throughout* as "light as a feather," and ready for the oven. *Therein* the heat volatizes the alcohol, which he excreted in the dough, and the result is that we have light and healthy, instead of heavy, unleavened, indigestible bread—but free from alcohol.

Now, wherever glucose (fruit or grape sugar) is found, this fermentation process caused by this living and breathing and propagating process of this yeast animal cell goes on, sooner or later. His food, when from grape juice, gives us wine; when from apples, cider; from pears, perry; from honey, metheglin; from malted grains, beer, etc. In all cases the starch or cane sugar is changed first to glucose, and then the yeast cell is set to work and secretes the alcohol by fermentation.

The process is, in short, this:

Glucose consists of Carbon (6 atoms, or equivalents), Hydrogen (12 atoms), Oxygen (6 atoms); or $C_6$, $H_{12}$, $O_6$. The yeast animal takes to feed and warm himself (assimilates) just one-half of this glucose, or $C_3$, $H_6$, $O_3$; of the other half he breathes off $C_1$, $O_2$, which is the formula of carbonic acid gas; and the rest of the glucose he excretes as *alcohol*, of which the chemical formula is $C_2$ $H_6$ $O_1$.

All alcohol comes at first in just this way and no other.

